# Identification of Risk Factors for the Development of Diabetic Retinopathy Among Palestinian Adults With Type 2 Diabetes Mellitus: A Cross‐Sectional Study

**DOI:** 10.1002/edm2.494

**Published:** 2024-06-14

**Authors:** Oadi N. Shrateh, Mohammad Abdelhafez, Suheir Ereqat, Lana Naser El Dein, Salam Iriqat

**Affiliations:** ^1^ Faculty of Medicine Al‐Quds University Jerusalem Palestine; ^2^ Department of Internal Medicine, Faculty of Medicine Al‐Quds University Jerusalem Palestine; ^3^ Biochemistry and Molecular Biology Department, Faculty of Medicine Al‐Quds University Jerusalem Palestine; ^4^ Diabetes Care Center Augusta Victoria Hospital Jerusalem Palestine; ^5^ Ocular Inflammatory Disease Department St John Eye Hospital Jerusalem Palestine

**Keywords:** diabetic retinopathy, Palestine, Type 2 diabetes mellitus

## Abstract

**Introduction:**

Although risk factors linked to diabetic retinopathy (DR) among patients with Type 2 diabetes mellitus (T2DM) have been extensively studied globally, the specific determinants of these factors in relation to DR in Palestine are presently not well understood.

**Methods:**

This retrospective cross‐sectional study included patients who underwent DR screening with a fundus camera (VersaCam *a*). The study included patients aged ≥18 with T2DM, excluding those with other types of diabetes or a history of malignancies. Univariable and multivariable logistic regressions were used to identify factors associated with DR.

**Results:**

A total of 1163 patients with T2DM were included in this study. Of these, 211 (18.1%) patients were classified in the DR group, 761 (65.4%) in the no DR group and 191 (16.4%) were ungradable. Among the included patients, 434 (37.3%) were male. A secondary level of education or higher and a BMI ≥30 kg/m^2^, compared with <25 kg/m^2^, were independently and inversely associated with DR, with odds ratios (ORs) of 0.46 (*p* < 0.001) and 0.58 (*p* = 0.046), respectively. A 5‐year increase in the duration of T2DM correlated with 45% higher odds of having DR (*p* < 0.001). Patients with DR were more likely to have HbA1c >7%, be physically inactive and use insulin, with ORs of 1.63 (*p* = 0.02), 2.05 (*p* < 0.001) and 1.53 (*p* = 0.03), respectively. Age, gender, occupational status, hypertension and hyperlipidaemia were not independent predictors of DR (*p* < 0.05).

**Conclusion:**

Longer duration of T2DM, HbA1c >7%, physical inactivity and insulin use were all independently associated with the presence of DR. Furthermore, a secondary or higher educational level and obesity demonstrated independent and inverse associations with the development of DR.

## Graphical abstract

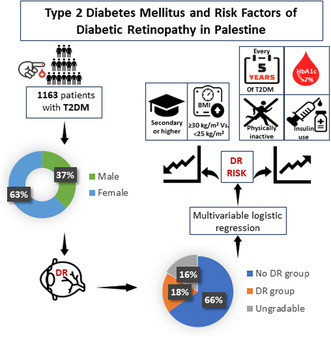

## 1 Introduction

Diabetic retinopathy (DR) remains the primary cause of legal blindness in individuals of working age [1]. DR is a prevalent microvascular complication of Type 2 diabetes mellitus (T2DM) and represents the leading cause of visual impairment among the elderly. This complication affects approximately 30%–40% of people with diabetes [2, 3]. In 2020, it was estimated that around 103 million adults worldwide were affected by DR, and this number could rise to 160 million by 2045. This substantial increase in cases is expected to have a significant impact on the prevalence of blindness and vision impairment, particularly among adults of working age groups [2, 4, 5].

The development of DR is directly related to a longer period of diabetes, uncontrolled hyperglycaemia and hypertension. Additionally, a higher and fluctuating HbA1c level is a significant factor contributing to the emergence and progression of DR [6, 7]. Additional risk factors encompass nephropathy, dyslipidaemia, smoking, earlier onset of diabetes and a higher body mass index (BMI); all previous factors are modifiable to prevent the progression of DR [8, 9, 10, 11]. Despite numerous epidemiologic studies and clinical trials exploring the primary risk factors for DR [12], there is significant variability in the consistency, pattern and strength of these risk factors.

Systematic screening and monitoring for DR have demonstrated their cost‐effectiveness in diminishing the occurrence of blindness and visual impairment in individuals with diabetes. Screening facilitates the precise timing of laser and medical treatments that can potentially halt the advancement of the disease [13]. The guidelines outlined by the World Health Organization (WHO) for DR screening recommend that patients with diabetes should undergo annual eye examinations [14].

The need for well‐designed and DR screening initiatives is becoming eminent. Therefore, the primary objective of this study was to assess the risk factors of DR among patients with T2DM in Palestine, introducing a handheld fundus camera (VersaCam *a*) for the first time in the country.

## 2 Materials and Methods

### 2.1 Study Participants and Data Collection

A retrospective cross‐sectional study was conducted at Augusta Victoria Hospital (AVH) in which the data were collected through a community outreach program during the period of 2021–2022. All data were collected using a dedicated data sheet, which was completed by a trained nurse. Herein, we reviewed the medical records of screened patients and included patients aged ≥18 with T2DM, regardless of the duration of diabetes mellitus. Patients with Type 1 diabetes mellitus, other types of diabetes or a current or previous history of malignancies were excluded. Overall, we enrolled 1163 patients in this study. The following data were collected: sociodemographic factors (sex, age, marital status, education and occupational levels and residential area), medical‐related factors (BMI, HbA1c, diabetes duration, cardiovascular diseases, hypertension and hyperlipidaemia), health behaviour–related factors (medication, smoking and physical activity) and diagnosis of DR.

### 2.2 Diagnosis of Diabetic Retinopathy

Retinopathy screening was conducted by a handheld fundus camera (VersaCam *a*) to determine the existence of DR, distinguishing between proliferative diabetic retinopathy (PDR) and nonproliferative diabetic retinopathy (NPDR) if present. Patients underwent screening using fundus photographs, which were then examined and classified by a skilled nurse from the AVH team. Briefly, eye drops were used to widen the pupils. Two coloured fundus photographs were taken during the examination to identify specific signs. Neovascularisation, vitreous haemorrhage or preretinal haemorrhage are indicative of PDR. Conversely, the presence of microaneurysms, dot haemorrhages, hard exudates or cotton wool spots without neovascularisation corresponds to NPDR. The absence of these signs in fundus photographs indicates the absence of DR. Using the Scottish grading protocol, patients with a grading of R1 or higher were identified as having retinopathy. In cases where the retinal images were deemed ungradable by the nurses, they were referred to an ophthalmologist for further examination.

### 2.3 Statistical Analysis

We conducted the statistical analysis using the R environment, version 4.1.3 (Vienna, Austria). Initially, we assessed continuous variables for normality using *Q*–*Q* plots and histograms. For descriptive statistics, we utilised the mean and standard deviation (SD) for normally distributed data or the median and interquartile range (IQR) for non‐normally distributed data. Categorical variables were expressed as frequencies and percentages. Subsequently, we compared the characteristics of the three study groups (DR, no diabetic retinopathy and ungradable) using analysis of variance or the Kruskal–Wallis test for continuous variables and the chi‐squared test or Fisher's exact test for categorical variables. We identified potential risk factors for DR using univariable logistic regression analysis, with DR status (patients with DR vs. patients without) as the dependent variable. Furthermore, we investigated the association of the following continuous variables: duration of DM, BMI and HbA1c with DR by categorising these variables, in line with previous studies [15, 16]. Additionally, we explored the ordinal variable categories (DM duration, BMI, education level and physical activity) for their association with DR using univariable logistic regression analysis. In each case, we estimated the effect of each category with the immediate lower category level used as a reference, independently of other variable categories. We constructed a multivariable logistic regression model to identify independent predictors of DR. Factors associated with DR at a significance level of *p* < 0.05 in univariable logistic regression were included in the model. BMI and HbA1c were treated as categorical variables, whereas age and DM duration were treated as continuous variables. Where appropriate, ordinal data were converted into dichotomous data on the basis of the exploratory univariable logistic regression findings to enhance clinical utility. The multivariable logistic regression model was further adjusted, as preplanned, to include known reported risk factors based on previous studies [5, 16, 17], regardless of the significance of the association. Associations with DR in univariable and multivariable logistic regressions were presented as odds ratios (ORs) with 95% confidence intervals (CI) along with the corresponding *p* values. We set the alpha level at 0.05.

## 3 Results

### 3.1 Study Participants

Clinical characteristics, demographics and anthropometric measures of study participants are indicated in Table 1. A total of 1163 patients with T2DM were eligible for the current study. The study participants were categorised into three groups on the basis of fundus photograph findings: 211 (18.1%) patients with DR, 761 (65.4%) without DR and 191 (16.4%) were classified as ungradable. The median age of all subjects was 62.1 years (IQR, 13), with 729 (62.7%) being female. Additionally, the median duration of diabetes mellitus was 11.1 years (IQR, 11), and the median BMI and HbA1c were 31.2 kg/m^2^ (IQR, 7) and 8% (IQR, 2.5), respectively. The majority of patients were from Hebron city (489, 42%), married (1002, 86.2%) and housewives (705, 60.6%), and they had varying educational backgrounds, with primary school education being the most common (381, 32.8%) as shown in Table 1.

**TABLE 1:** Demographic characteristics of study participants.

Variable	All patients (*n* = 1163)	No diabetic retinopathy (*n* = 761)	Diabetic retinopathy (*n* = 211)	Ungradable (*n* = 191)	*p*
Sociodemographic factors					
Age (year)	62.1 [13]	60.1 [13]	63.1 [11]	64.1 [12]	<0.001
Gender (male)	434, 37.3	267, 35.1	87, 41.2	80, 41.9	0.095
City *n*, %					
Hebron	489, 42	320, 42	76, 36	93, 48.7	0.017^a^
Bethlehem	451, 38.8	294, 38.6	89, 42.2	68, 35.6	
Jerusalem	67, 5.8	53, 7	8, 3.8	6, 3.1	
Ramallah	127, 10.9	81, 10.6	28, 13.3	18, 9.4	
Qalqilya	29, 2.5	13, 1.7	10, 4.7	6, 3.1	
Education level *n*, %					
Illiterate	102, 8.8	56, 7.4	31, 14.7	15, 7.9	<0.001
Primary	381, 32.8	222, 29.2	86, 40.8	73, 38.2	
Secondary	264, 22.7	187, 24.6	38, 18	39, 20.4	
High school	250, 21.5	184, 24.2	35, 16.6	31, 16.2	
Bachelor's degree or higher	166, 14.3	112, 14.7	21, 10	33, 17.3	
Marital status *n*, %					
Married	1002, 86.2	668, 87.8	179, 84.8	155, 81.2	0.208^a^
Single	20, 1.7	11, 1.4	5, 2.4	4, 2.1	
Divorced	140, 12	81, 10.6	27, 12.8	32, 16.8	
Widow	1, 0.1	1, 0.1	0, 0	0, 0	
Occupational status *n*, %					
Housewife	705, 60.6	477, 62.7	120, 56.9	108, 56.5	0.006^a^
Student	2, 0.2	1, 0.1	0, 0	1, 0.5	
Employed	209, 18	144, 18.9	35, 16.6	30, 15.7	
Unemployed	41, 3.5	29, 3.8	4, 1.9	8, 4.2	
Retired	206, 17.7	110, 14.5	52, 24.6	44, 23	
Medical‐related factors					
Duration of DM (year)	11.1 [11]	8.1 [8]	16.1 [11]	14.1 [10]	<0.001
DM duration categories *n*, %					
<5 years	275, 23.6	231, 30.4	15, 7.1	29, 15.2	<0.001
5–10 years	292, 25.1	221, 29	39, 18.5	32, 16.8	
10–15 years	248, 21.3	156, 20.5	50, 23.7	42, 22	
15–20 years	182, 15.6	86, 11.3	52, 24.6	44, 23	
>20 years	166, 14.3	67, 8.8	55, 26.1	44, 23	
BMI (kg/m^2^)	31.2 [7]	31.3 [7.4]	30.1 [6.8]	31.2 [6]	0.202
BMI categories *n*, %					
<25 kg/m^2^	118, 10.1	72, 9.5	27, 12.8	19, 12.8	0.253
25–29.9 kg/m^2^	360, 31	277, 29.8	74, 35.1	59, 30.9	
≥30 kg/m^2^	685, 58.9	462, 60.7	110, 52.1	113, 59.2	
HbA1c (%)	8 [2.5]	7.9 [2.7]	8.5 [2.8]	8 [3]	<0.001
HbA1c categories *n*, %					
HbA1c ≤7%	388, 33.4	281, 36.9	44, 20.9	63, 33	<0.001
HbA1c >7%	775, 66.6	480, 63.1	167, 79.1	128, 67	
Hypertension disease *n*, %	728, 62.6	457, 60.1	145, 68.7	126, 66	0.041
Cardiovascular diseases *n*, %	119, 10.2	70, 9.2	24, 11.4	25, 13.1	0.237
Hyperlipidaemia *n*, %	397, 34.1	244, 32.1	81, 38.4	72, 37.7	0.121
Health behaviour–related factors					
Smoking *n*, %	173, 14.9	114, 15	31, 14.7	28, 14.7	>0.99
Physical activity *n*, %					
Inactive	233, 20	124, 16.3	67, 31.8	42, 22	<0.001
<150 min/week	793, 68.2	539, 70.8	124, 58.8	130, 68.1	
150–300 min/week	137, 11.8	98, 12.9	20, 9.5	19, 9.9	
Medications *n*, %					
Insulin	426, 36.6	223, 29.3	115, 54.5	88, 46.1	<0.001
Oral antidiabetic medications	1080, 92.9	717, 94.2	196, 92.9	167, 87.4	0.005

*Note:* Continuous variables are presented as median [interquartile range], whereas categorical variables are expressed as frequencies and percentages. *p* values were obtained using the Kruskal–Wallis test for continuous variables and the chi‐squared test for categorical variables.

Abbreviations: BMI, body mass index; DM, diabetes mellitus; HbA1c, glycated haemoglobin.

^a^ *p* value was obtained through the Fisher exact test. *p* value <0.05 is considered significant.

### 3.2 Risk Factors for Diabetic Retinopathy

On the basis of the univariable logistic regression analysis, several factors have shown significant associations with DR. Older age, being retired, longer diabetes duration, higher HbA1c levels, having hypertension and being on insulin therapy were significantly associated with higher odds of DR (*p* < 0.05; Table 2), whereas patients with a secondary school level of education or higher, a BMI ≥30 kg/m^2^ compared with <25 kg/m^2^, and those who are physically active, as opposed to inactive, showed a significantly lower odds of having DR (*p* < 0.05). On the contrary, the marital status, history of cardiovascular diseases, hyperlipidaemia and receiving oral hypoglycaemic medications did not exhibit a significant association with the risk of DR.

**TABLE 2:** Univariable logistic regression analysis to identify factors associated with diabetic retinopathy.

Variable	COR (95% CI)	*p*
Sociodemographic factors		
Age (per year)	1.04 (1.02–1.06)	<0.001
Gender (male)	1.30 (0.95–1.77)	0.101
Education level		
Illiterate	1 (ref)	—
Primary	0.70 (0.42–1.16)	0.166
Secondary	0.37 (0.21–0.64)	<0.001
High school	0.34 (0.19–0.61)	<0.001
Bachelor's degree or higher	0.34 (0.18–0.64)	<0.001
Marital status		
Married	1 (ref)	—
Single	1.70 (0.58–4.94)	0.333
Divorced	1.24 (0.78–1.98)	0.358
Widow	0.00 (0.00–NA)	0.982
Occupational status		
Employed	1 (ref)	—
Student	0.00 (0.00–NA)	0.982
Housewife	1.04 (0.68–1.58)	0.872
Unemployed	0.57 (0.19–1.72)	0.317
Retired	1.94 (1.19–3.19)	0.009
Medical‐related factors		
Duration of DM (per year)	1.10 (1.08–1.12)	<0.001
DM duration (per 5 years)	1.62 (1.45–1.80)	<0.001
DM duration categories		
<5 years	1 (ref)	—
5–10 years	2.72 (1.46–5.07)	0.002
10–15 years	4.94 (2.68–9.10)	<0.001
15–20 years	9.31 (4.98–17.41)	<0.001
>20 years	12.64 (6.72–23.79)	<0.001
BMI (kg/m^2^)	0.98 (0.95–1.004)	0.088
BMI categories		
<25 kg/m^2^	1 (ref)	—
25–29.9 kg/m^2^	0.87 (0.52–1.45)	0.594
≥30 kg/m^2^	0.63 (0.39–1.04)	0.069
HbA1c (%)	1.14 (1.06–1.22)	<0.001
HbA1c categories		
HbA1c ≤7%	1 (ref)	—
HbA1c >7%	2.22 (1.55–3.20)	<0.001
Hypertension disease	1.46 (1.06–2.02)	0.022
Cardiovascular diseases	1.27 (0.78–2.07)	0.345
Hyperlipidaemia	1.32 (0.96–1.81)	0.085
Health behaviour–related factors		
Smoking	0.98 (0.64–1.50)	0.917
Physical activity		
Inactive	1 (ref)	—
<150 min/week	0.43 (0.30–0.61)	<0.001
150–300 min/week	0.38 (0.21–0.66)	<0.001
Medications		
Insulin	2.89 (2.11–3.95)	<0.001
Oral antidiabetic medications	0.80 (0.44–1.47)	0.476

*Note:* Odds ratios and *p* values were obtained from logistic regression analysis with diabetic retinopathy (vs. no diabetic retinopathy) as the dependent variable. *p* value <0.05 is considered significant.

Abbreviations: BMI, body mass index; CI, confidence interval; COR, crude odds ratio; DM, diabetes mellitus; HbA1c, glycated haemoglobin; ref, reference; vs., versus.

To investigate the influence of transitioning between ordinal variable categories on the risk of DR, we conducted an exploratory univariable logistic regression analysis in which we compared each category level with the immediately lower one, as illustrated in Table 3. We found that the transition from primary to secondary school education level showed a significant reduction in the odds of having DR, with an OR of 0.53 (95% CI, 0.34–0.81), *p* = 0.003. Additionally, for each 5‐year increase in the duration of DM, there was a significantly higher chance of developing DR, except for patients with a DM duration of ≥20 years, who did not exhibit a significant difference compared with patients having DM for 15–20 years (OR, 1.36; 95% CI, 0.83–2.23; *p* = 0.227). Compared with overweight individuals, obese patients showed a marginally significant difference (*p* = 0.066) with a lower likelihood of having DR (OR, 0.73; 95% CI, 0.52–1.02). Among physically active patients, the level of physical activity did not demonstrate a significant impact on the development of DR (OR, 0.89; 95% CI, 0.53–1.49; *p* = 0.651).

**TABLE 3:** Univariable logistic regression analysis to outline the impact of ordinal variables category transitions on the risk of diabetic retinopathy.

Variable^a^	COR (95% CI)	*p*
Education level		
Primary (vs. illiterate)	0.70 (0.42–1.16)	0.166
Secondary (vs. primary)	0.53 (0.34–0.81)	0.003
High school (vs. secondary)	0.94 (0.57–1.55)	0.797
Bachelor's degree or higher (vs. high school)	0.99 (0.55–1.78)	0.962
DM duration categories		
5–10 years (vs. <5 years)	2.72 (1.46–5.07)	0.002
10–15 years (vs. 5–10 years)	1.82 (1.14–2.90)	0.012
15–20 years (vs. 10–15 years)	1.89 (1.18–3.02)	0.008
>20 years (vs. 15–20 years)	1.36 (0.83–2.23)	0.227
BMI categories		
25–29.9 kg/m^2^ (vs. <25 kg/m^2^)	0.87 (0.52–1.45)	0.594
≥30 kg/m^2^ (vs. 25–29.9 kg/m^2^)	0.73 (0.52–1.02)	0.066
Physical activity		
<150 min/week (vs. inactive)	0.43 (0.30–0.61)	<0.001
150–300 min/week (vs. <150 min/week)	0.89 (0.53–1.49)	0.651

*Note:* Odds ratios and *p* values were obtained from logistic regression analysis with diabetic retinopathy (vs. no diabetic retinopathy) as the dependent variable. *p* value <0.05 is considered significant.

Abbreviations: BMI, body mass index; CI, confidence interval; COR, crude odds ratio; DM, diabetes mellitus; vs., versus.

^a^ The effect of each category within the variable was determined separately, with the immediate lower category level used as a reference.

### 3.3 Identification of Independent Predictors for Diabetic Retinopathy

To identify the independent predictors of DR, we conducted a multivariable logistic regression analysis, adjusting for all factors that showed a significant association with DR development in the univariable logistic regression. As predetermined, the model was additionally adjusted for gender, cardiovascular diseases and hyperlipidaemia. Moreover, the educational level and physical activity, based on the findings from the univariable logistic regression in Tables 2 and 3, were treated as dichotomous variables. As shown in Table 4, both a secondary school or higher education level and obesity were independently associated with a lower likelihood of having DR, with adjusted ORs of 0.46 (95% CI, 0.32–0.65; *p* < 0.001) and 0.58 (95% CI, 0.34–0.99; *p* = 0.046). The duration of DM was independently associated with the development of DR. We found that for every 5‐year increase in DM duration, the odds of having DR increased by 45% (95% CI, 1.27–1.64; *p* < 0.001). Furthermore, patients with DR were more likely to have HbA1c levels >7%, be physically inactive and receive insulin; all of these factors independently predicted the presence of DR. Our results revealed that patients with DR were twice as likely to be physically inactive, with an adjusted OR of 2.05 (95% CI, 1.39–3.03), *p* < 0.001. The multivariable analysis revealed no correlation between age, retirement and hypertension with DR, even though this association was observed in the univariable analysis (Table 4).

**TABLE 4:** Multivariable logistic regression model to identify independent predictors of diabetic retinopathy.

Variable	Adjusted OR (95% CI)	Adjusted *p* value
Age (per year)	1.01 (0.98–1.03)	0.508
Gender (male)	1.12 (0.32–3.92)	0.863
Education level		
Secondary school or higher education	0.46 (0.32–0.65)	**<0.001**
Occupational status		
Employed	1 (ref)	—
Student	0.00 (0.00–NA)	0.982
Housewife	0.77 (0.21–2.75)	0.685
Unemployed	0.55 (0.17–1.78)	0.316
Retired	1.29 (0.73–2.28)	0.371
DM duration (per 5 years)	1.45 (1.27–1.64)	**<0.001**
BMI categories		
<25 kg/m^2^	1 (ref)	—
25–29.9 kg/m^2^	0.81 (0.46–1.42)	0.462
≥30 kg/m^2^	0.58 (0.34–0.99)	**0.046**
HbA1c categories		
HbA1c >7% (vs. ≤7%)	1.63 (1.08–2.45)	**0.02**
Hypertension disease	1.22 (0.83–1.78)	0.305
Cardiovascular diseases	0.97 (0.55–1.71)	0.913
Hyperlipidaemia	1.12 (0.77–1.62)	0.56
Physical activity		
Inactive (vs. active)	2.05 (1.39–3.03)	**<0.001**
Medications		
Insulin (yes)	1.53 (1.04–2.24)	**0.03**

*Note:* Odds ratios and *p* values were obtained from multivariable logistic regression analysis with diabetic retinopathy (vs. no diabetic retinopathy) as the dependent variable. *p* value <0.05 is considered significant. *p* values below the significance level are highlighted in bold.

Abbreviations: BMI, body mass index; CI, confidence interval; DM, diabetes mellitus; OR, odds ratio; ref, reference; vs., versus.

## 4 Discussion

Diabetic retinopathy has been reported as the most prevalent complication accounting for approximately 17.3% among Palestinian diabetic patients [18]. The primary factor contributing to the risk of DR is primarily linked to inadequate glycaemic control and the duration of diabetes. A recent study conducted in the northern provinces of Palestine, employing a cross‐sectional approach, found that the incidence of DR was 30%, with 12.2% of cases classified as proliferative DR. The study also identified hypertension, uncontrolled T2DM and the duration of diabetes as the key factors associated with the development of DR. Furthermore, the duration of diabetes emerged as a significant predictor for the occurrence of proliferative DR [1]. Herein, we conducted a retrospective cross‐sectional study to investigate the risk factors of DR among 1163 patients with T2DM in Palestine. Our study showed that an extended duration of T2DM, HbA1c levels exceeding 7%, lack of physical activity and the use of insulin were all independently linked to the occurrence of DR. Previous studies have demonstrated a correlation between the development of DR and an extended duration of diabetes, poorly controlled hyperglycaemia and hypertension. Moreover, elevated and erratic levels of HbA1c play a noteworthy role in the initiation and progression of DR indicating that prolonged exposure to hyperglycaemia contributes to microvascular complications [6, 7]. Other contributing risk factors include nephropathy, dyslipidaemia, smoking, an earlier onset of diabetes and a higher BMI have been reported. Importantly, these factors can be altered to mitigate the advancement of DR [8, 9, 10, 11]. Recent investigations have highlighted a positive link between physical activity and both the onset and severity of DR. However, the specific type and frequency of physical activity necessary to exert a significant impact on DR have not been precisely determined [12, 19].

A previous study in Palestine showed that the probability of a patient with ≥20 years of diabetes to develop PDR is seven times higher than other patients with a diabetes duration of 5–10 years [20]. Another Palestinian study also found that those aged 40 years and more had double the likelihood of obtaining DR in comparison with their younger counterparts [21]. A retrospective study enrolled 373 patients with diabetes reported several factors contributing to the development of DR, including older age, male gender, a longer duration of diabetes, receiving only insulin therapy and high systolic blood pressure. A notable correlation was observed between an extended duration of T2DM and the occurrence of DR [22]. Consistent results supporting this association were identified in various other studies [15, 23, 24, 25, 26], all of which regarded prolonged diabetes duration as a predictive factor for the development of DR [27]. In comparison, our analysis identified several demographic and clinical factors associated with the development of DR. Diabetes duration and insulin therapy were found to be significant risk factors, aligning with the results reported in aforementioned studies. These associations are consistent with other previous studies, such as those carried out in the Indian population, by which Raman et al. and Khan et al. found that insulin was a risk factor for DR in different age‐of‐onset groups [28, 29]. In consistent with our findings, several studies showed no association between gender and the occurrence of DR [15, 30, 31]. In contrast, other studies revealed an increase in the incidence of DR in male patients [26, 30].

On the contrary, our study identified a significant association between educational level and DR, indicating that patients with a secondary school education or higher had a lower likelihood of developing DR compared with those with a lower educational level. In line with our results, Dweib and El Sharif [21] showed that Palestinian individuals lacking basic literacy skills exhibited a higher likelihood of receiving a retinopathy diagnosis than those with a secondary education, diploma or college education. Similar findings have been reported in studies conducted in Malaysia and India, suggesting that higher educational attainment may be protective against DR [32, 33]. A study conducted on 496 Ethiopian participants revealed that individuals with an educational level of diploma and above are at a significantly lower risk of developing DR than those with lower educational levels [34]. This emphasises the importance of considering socioeconomic factors in the context of diabetic complications.

Moreover, several studies revealed no correlation between BMI and the onset of DR [15, 35], whereas others indicated that higher BMI was positively associated with DR [36, 37, 38]. Inverse relationship between BMI and DR severity was also reported in Asian patients with T2DM, in which a higher BMI appeared to confer a protective effect on DR, whereas higher waist‐to‐hip ratio (WHR) was associated with the presence and severity of DR in women [39]. Our results indicated that BMI is a significant factor associated with the risk of DR.

Considerable evidence underscored the connection between physical inactivity and the onset and complications of diabetes. However, there is limited knowledge regarding the relationship between physical inactivity and DR in terms of its onset, progression and severity. Certain health professionals have integrated physical activity as a crucial component of disease prevention and management [19]. Despite robust evidence supporting the relationship between physical activity and disease status, a significant proportion of the global population failed to meet the minimum recommended levels of physical activity outlined by organisations such as the American College of Sports Medicine and other Public Health and Exercise Authorities [40, 41]. Recent studies have brought attention to the positive association between physical activity and both the onset and severity of DR. Nevertheless, the specific type and frequency of physical activity required to have a significant impact on DR have not been precisely determined [19]. A study by Aro et al. investigated whether the implementation of lifestyle changes early in the onset of diabetes would yield favourable effects on the occurrence of DR. Their findings indicated a positive impact of incorporating lifestyle changes into diabetes management plans, specifically in delaying the progression or onset of DR [42, 43]. Beijing Eye Study conducted on 3468 participants showed that a higher level of physical activity was linked to a reduced prevalence of DR [44]. A review of meta‐analyses conducted in 2022 revealed that sedentary behaviour was linked to an increased risk of DR [45]. Consistently, our study confirmed the protective effect of being physically active against the development of DR compared with those who are inactive.

The optimal HbA1c cut‐off for detecting any DR was identified as 49 mmol/mol (6.6%), whereas for moderate or severe retinopathy, the cut‐off was determined to be 52 mmol/mol (6.9%) [46]. Our study also showed that the high levels of HbA1c are associated with an increased risk of DR occurrence specifically those with levels above the cut‐off of 7%. Another Palestinian study detected that the glycaemic control as indicated by the median of the HbA1c level was also associated with PDR [20]. Hsu et al. concluded that the development or progression of DR in T2DM may be influenced by short‐term glycaemic fluctuation, whereas long‐term glycaemic fluctuation, as indicated by HbA1c levels, seems to play a more significant role in the occurrence of retinopathy in patients [47]. Another study verified that the suggested HbA1c threshold of 48 mmol/mol (6.5%) was effective in accurately detecting DR [46].

In the current study, univariable analysis showed that occupational status has a significant impact on the risk of DR in which retirement increases the chance of DR development. However, this was not evident in the multivariable analysis. Lee [48] observed that individuals with occupations tend to undergo more frequent and dedicated screening processes, indirectly leading to a reduced risk of DR. Similarly, Klein et al. [49] found that employed individuals have a lower risk of DR development compared with nonemployed patients.

The role of dyslipidaemia in DR has gained increased attention, although findings remain contentious. Previous studies have indicated an association between dyslipidaemia and DR, yet the causal effect remains uncertain. Ereqat et al. [20] revealed that the odds for patients with dyslipidaemia to get PDR were 2.74 times higher than patients with NPDR. Yau et al. [2] discovered a link between higher total serum cholesterol and an increased risk of diabetic macular oedema, emphasising the significance of dyslipidaemia, hyperglycaemia and hypertension as major modifiable risk factors for all types of DR. Another study provided robust evidence supporting the notion that dyslipidaemia promotes the development of DR through increased secretion of vascular endothelial growth factors A, C and D, as well as placental growth factor in patients with DR [50]. Additionally, elevated lipids are linked to endothelial dysfunction, resulting in hemodynamic changes, retinal tissue hypoxia, microcirculatory disorders and the breakdown of the blood–retinal barrier, contributing to the development of DR [51]. In contrast, Wong et al. [52] presented conflicting reports, suggesting that higher total cholesterol levels were associated with reduced odds of DR. Although our results did not show a clear causal relationship between hyperlipidaemia and DR, the mechanism behind them still deserves further investigation.

Although some studies demonstrated that persistent and heavy smoking increases the risk of microvascular complications, including nephropathy and neuropathy, their findings did not provide clear and interpretable evidence regarding the association between smoking and retinopathy [53]. Additionally, the majority of studies showed that smoking is not likely to be an important risk factor for DR [30, 35, 54, 55]. However, a previous Palestinian study showed that smoking status is marginally associated with the severity of DR [20]. In contrast, the findings of this study demonstrated no association between smoking status and the development of DR.

In Palestine, the community‐based screening process for DR faces numerous challenges and obstacles, like checkpoints that hinder the accessibility and reachability of people to primary care centres. Despite these difficulties, implementing outreach programs and utilising the fundus cameras could significantly enhance health outcomes, especially for those who residing in neglected areas. The robustness of this study is affirmed by the substantial number of participants and the representation of the West Bank population, encompassing patients from different districts. The findings of our study hold potential utility for healthcare practitioners in Palestine, as they can aid in the early detection and screening of individuals at a high risk of developing DR.

On the contrary, it is important to recognise that the present study has certain limitations. These limitations arise from the absence of data concerning other diabetic complications, including nephropathy and neuropathy. The fundus camera used to determine the status of DR was unable to confirm the diagnosis in 191 (16.4%) participants. These patients were referred to an ophthalmologist for follow‐up, and their final diagnosis data are not available. Additionally, we did not investigate factors related to the severity of DR, as data on DR grade and severity were not available for the majority of the studied patients.

In conclusion, this study emphasises particular significance of DR and its correlation with several crucial factors that could potentially expedite the development of DR in patients diagnosed with T2DM within Palestinian community. Our study indicated that longer duration of T2DM, HbA1c >7%, physical inactivity and insulin use were all independently associated with the presence of DR. Furthermore, a secondary or higher educational level and obesity demonstrated independent and inverse associations with the development of DR. Such identification can contribute to the improvement of patient monitoring and the reduction in disease exacerbation. Additional studies are needed to declare these associations obviously and to assess the awareness of DR among patients with diabetes.

## Author Contributions

O.N.S., M.A., S.E., L.N.E.D. and S.I. conceptualized the study; L.N.E.D. and S.I. were responsible for resources; S.E. and S.I. were responsible for project administration and supervision; M.A. was responsible for data curation, and formal analysis; L.N.E.D. and S.I. were responsible for investigation; M.A. and S.E. were responsible for methodology; S.E. and S.I. were responsible for validation, and visualization; O.N.S. wrote the original draft; O.N.S., M.A., S.E., L.N.E.D. and S.I. reviewed and edited the manuscript.

## Ethics Statement

Informed consent were received from all participants before starting the work. The study procedure was approved by the Research Ethics Committee of Augusta Victoria Hospital (AVH) with reference number of AVH‐2023.

## Consent

Written consent was obtained from the participants regarding publishing this research in accordance with the journal's patient consent policy.

## Conflicts of Interest

The authors declare no conflicts of interest.

## Data Availability

The data that support the findings of this study are available from the corresponding author upon reasonable request.
